# Ventilator-associated pneumonia by *Weeksella virosa*: case report

**DOI:** 10.1186/s12879-023-08927-0

**Published:** 2024-01-02

**Authors:** Luis Alberto de la Fuente García Peña, Alan Ulises Mendoza García, Josué Eli Villegas-Dominguez, Félix Guillermo Márquez Celedonio, Hugo Arana Vidal, Karime Azuara Díaz

**Affiliations:** 1 Facultad de Medicina, Universidad del Valle de Mexico, Campus Veracruz, Veracruz, Mexico; 2 High Specialty Regional Hospital B, ISSSTE Veracruz, Veracruz, Mexico

**Keywords:** Pneumonia, Mechanical ventilator, Weeksella virosa, Nosocomial infection

## Abstract

**Background:**

Weeksella virosa pneumonia is an infection that has been described as a healthcare-associated infection. This is a rare gram-negative anaerobic bacterium associated with the use of mechanical ventilation for a long period of time and is more frequent in immunosuppressed patients. This is the first case reported in the state of Veracruz and the second in Mexico.

**Case presentation:**

We present the case of a 64-year-old female from Veracruz, Mexico who developed an infectious process in the right pelvic limb after a transcatheter aortic valve replacement procedure and subsequently developed sudden cardiorespiratory arrest requiring mechanical ventilation, with subsequent imaging studies demonstrating a pneumonic process associated with a nosocomial infection.

**Discussion and Conclusions:**

We should take into consideration that this pathogen affects not only adults with multiple comorbidities but also children with renal, hepatic, or oncological pathologies, as well as immunocompromised patients, who should be considered high-risk populations for W. virosa infection.

## Introduction

**Fig. 1:**
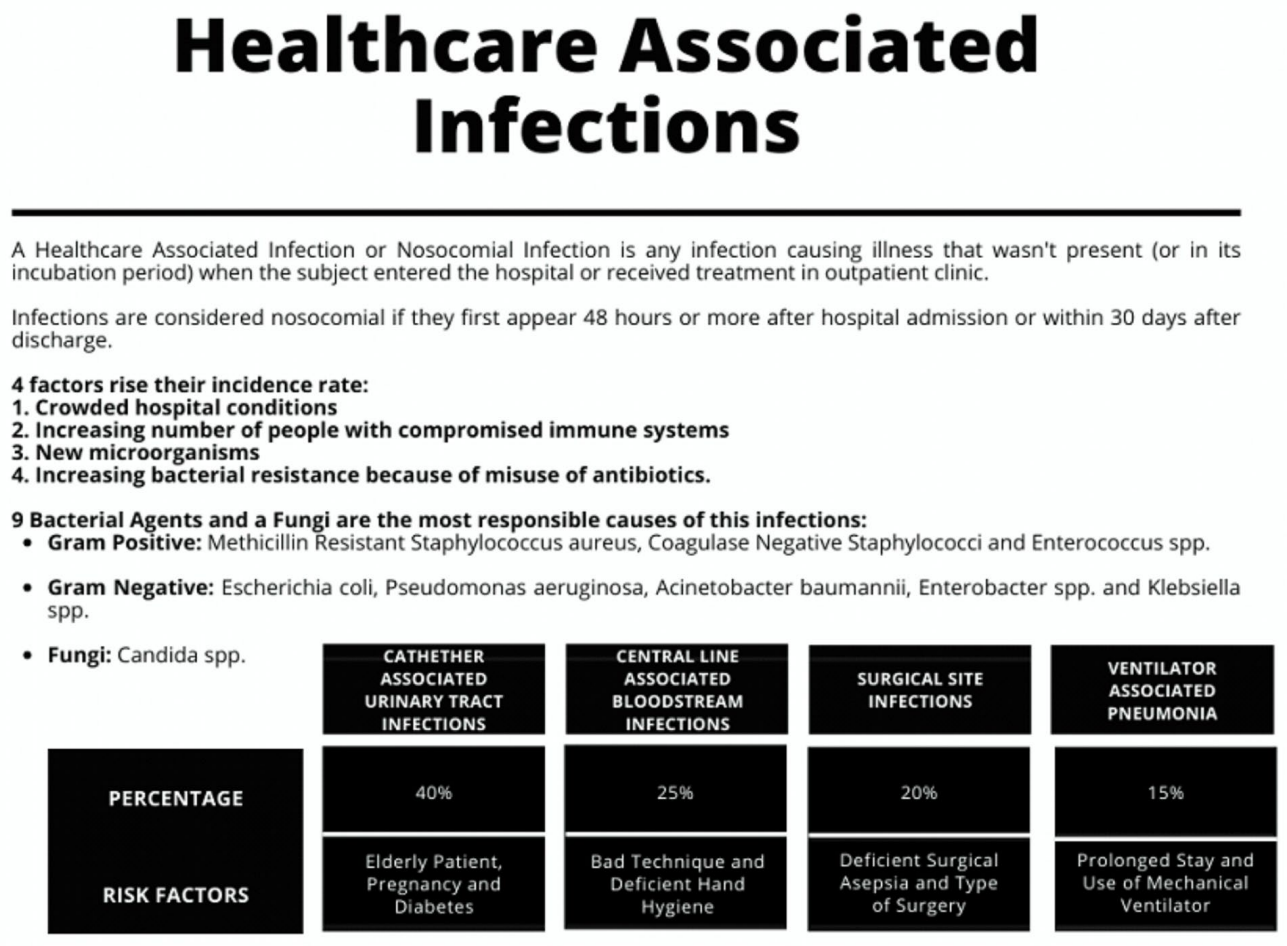
Prevalence of the different types of health care associated infections and their main risk factors

Health Care Associated Infections (HCAIs), also known as nosocomial or in-hospital infections, are defined as infections acquired by a patient during treatment in a hospital or other health facility, excluding the possibility that the patient had or was incubating the pathogen at the time of admission. This definition also includes patients who have been discharged and occupational infections contracted by health personnel. (Fig. 1) [1].

The risk of acquiring such an infection is 5 to 10%, with a higher risk in Intensive Care Unit (ICU) personnel, reaching up to 20–40% [2]. The prevalence of mechanical ventilation-associated pneumonia is 5–50% in patients who require mechanical ventilation for more than 48 h, with an average time of development of 5–9 days of mechanical ventilation [3, 4].

Among the most frequent agents are gram-positive bacteria such as methicillin-resistant *Staphylococcus aureus* and *Streptococcus pneumoniae* and gram-negative bacteria such as *Pseudomonas aeruginosa*, Acinetobacter baumanni, Enterobacteria (*Escherichia coli* and *Klebsiella pneumoniae*) and *Haemophilus influenzae*, although new microorganisms such as W. virosa have recently been reported as etiologic agents [1, 4].

Weeksella virosa is an uncommon gram-negative aerobic bacterium, first described in 1970 by Pickett and Manclark as a nonsaccharolytic flavobacteria and identified according to Gram staining as negative in the form of bacillus, which can be grown in chocolate agar and blood agar after 48 h incubation at 22, 35 and 42 °C [5–7]. It must not be confused with Bergeyella zoohelcum (formerly part of the genus Weeksella), which is associated with infection from animal bites [8]. The difference between the two species is that B. zoohelcum is urease positive and has an innate resistance to polymyxin [8–11].

This microorganism is usually detected and isolated when susceptibility tests are performed in the group of other non-Enterobacteriaceae gram-negative bacilli [5]. It is clinically associated with bacteremia, peritonitis and urinary infections in immunocompromised patients and nosocomial infections [12]. It is more prevalent in female patients and patients with comorbidities such as kidney disease, obesity, hepatopathy and diabetes, as well as those in intensive care units [13, 14].

The few occasions in which this organism has been isolated have been in urine samples (43%), cervical exudates (14%) and vaginal exudates (16%) [12, 13, 15, 16]. However, it has been isolated twice from blood and spinal fluid samples. Mardy et al. reported that of the vaginal exudate samples, a 2% incidence was found in asymptomatic patients or those presenting some symptom of vaginal infection [16]. Interestingly, a group in an English prison reported an incidence of 15% in patients who had a high risk of sexually transmitted diseases [16].

It is important to consider this bacteria if a gram-negative aerobic bacillus grows after 36 to 48 h of incubation in cultures of blood, sputum, urine, or peritoneal fluid [12, 16, 14, 17–20]. Once the pathogen is isolated, the empirical use of piperacillin, aztreonam or a carbapenem antibiotic is recommended. TMP-SMX, ciprofloxacin and aminoglycosides should not be used unless an antibiogram showing susceptibility is available [16, 21].

**Fig. 2:**
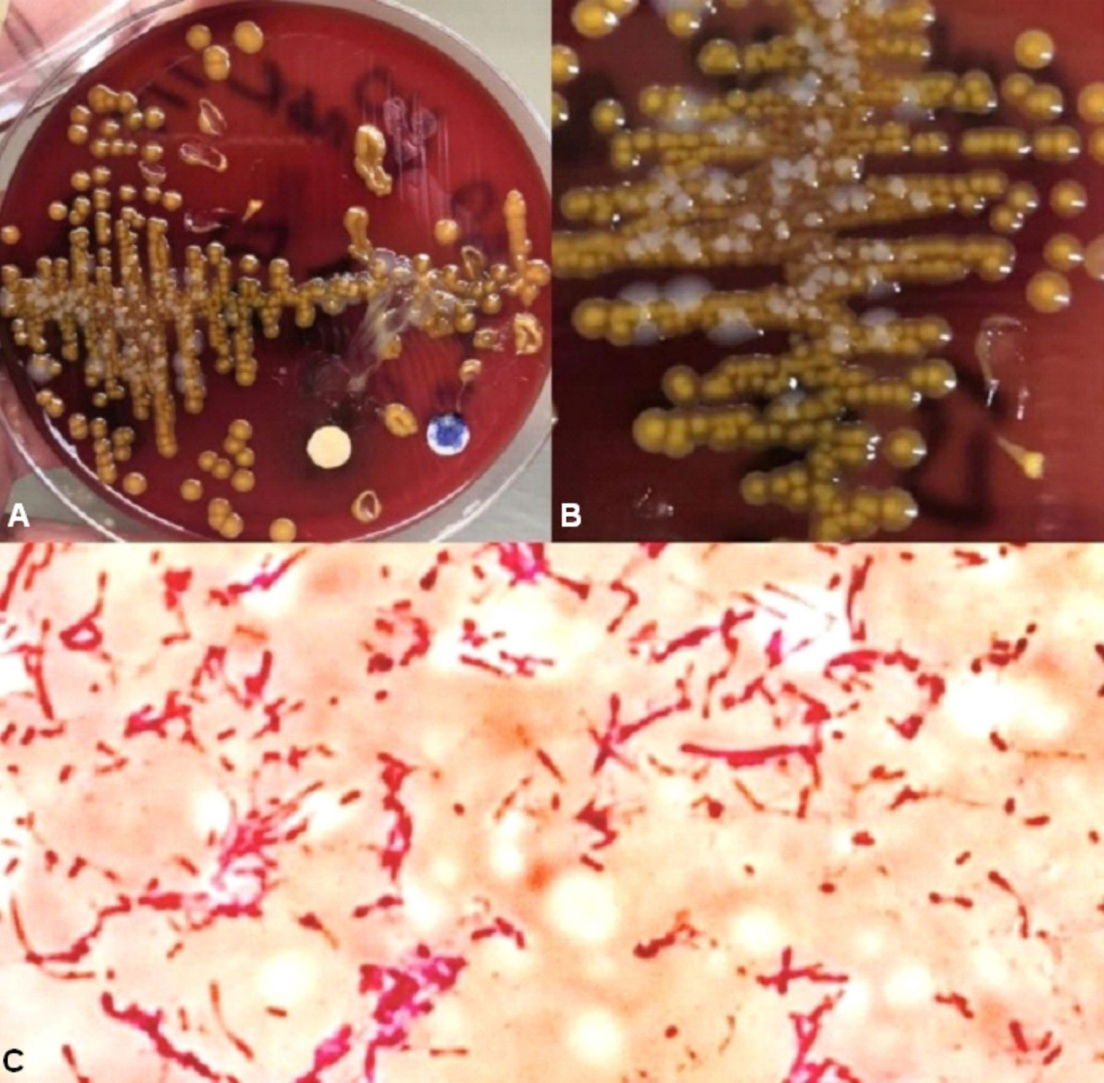
(**A** and **B**) Blood agar culture showing bacterial colonies with an extremely mucous cream color with an orange/yellowish pigment. (**C**) Microscope view of these colonies where the presence of Gram-Negative Bacilli is observed

This organism has the particularity of not growing in MacConkey agar, and the culture usually has an extremely mucous and cream color with an orange/yellowish pigment [6, 7, 9, 10]. Biochemically, the organism is positive for oxidase, indole and catalase (Fig. 2) [6, 7]. In vitro susceptibility studies report the effectiveness of the following antibiotics against this microorganism: piperacillin, monobactam, cephalosporins, fluoroquinolones and carbapenems [5]. Resistance has been noted in vitro with the use of aminoglycosides, nalidixic acid and nitrofurantoin.

## Case presentation

We present the case of a 64-year-old female patient from Soledad de Doblado, Veracruz, who was referred to our hospital in a coma on mechanical ventilation without sedation with broad-spectrum antimicrobial treatment. Seven days before her arrival, in another hospital, a transcatheter aortic valve implantation (TAVI) procedure was performed due to a history of aortic stenosis, severe aortic insufficiency, and KDIGO III renal chronic disease. During the postoperative period, she presented cardiac arrest, requiring advanced resuscitation maneuvers with airway management, and three cycles of cardiopulmonary resuscitation were given before returning to spontaneous circulation. She was then admitted to the ICU under sedation, invasive mechanical ventilation, antibiotics and thromboprophylaxis. Upon admission to our hospital, a skull CT scan was performed with no evidence of injury. An electroencephalogram was also performed, which was reported as compatible with ischemic encephalopathy, considered probable brain death.

At physical examination, the patient was dependent on vasoactive amines, with a temporary pacemaker, on mechanical ventilation, and without sedation. The pupils were myotic and unresponsive to luminous or external stimuli, and a 3-point Glasgow Coma Scale at pulmonary auscultation showed bilateral crackling rales on both lung bases.

**Fig. 3:**
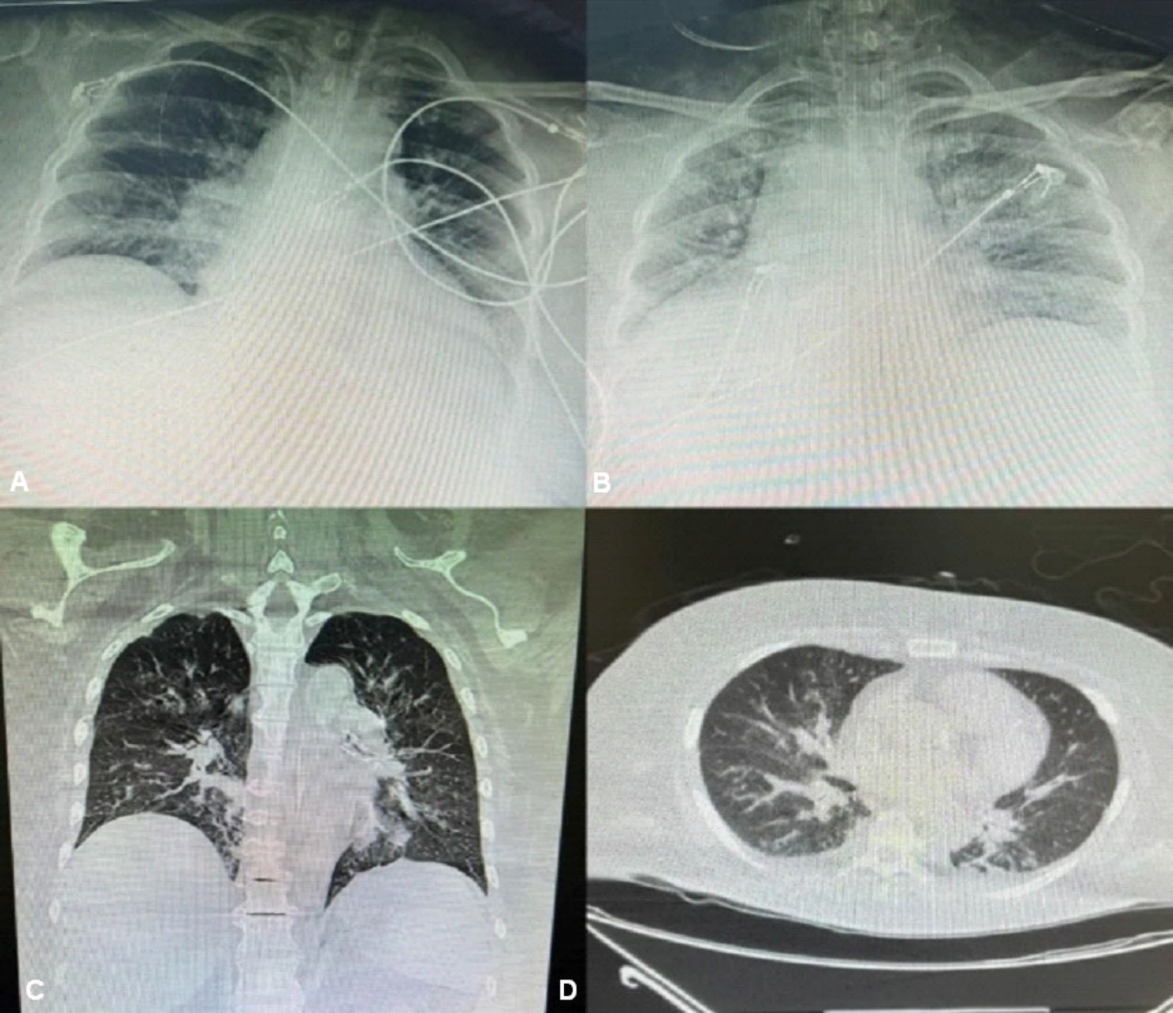
X-rays and CT scans showing pulmonary consolidations and pleural effusions suggesting a pneumonic process

Paraclinics performed at admission are reported: gasometry values suggesting metabolic acidosis with added respiratory alkalosis and leukocytosis of 17,000 with predominance of polymorphonuclear cells. A chest radiograph was obtained and demonstrated the presence of a pneumonic process with basal pulmonary consolidations. A subsequent simple chest CT was performed, which demonstrated bilateral pleural effusion (Fig. 3). Bronchoalveolar lavage was performed, and collected samples were placed on blood agar, chocolate agar and MacConkey agar. Gram-negative bacilli were isolated on blood agar, which were later identified using the VITEK technique and PCR sequencing as oxidase and catalase positive and determined by results such as Weeksella virosa.

When this culture was subjected to the Kirby-Bauer method and MIC bacterial susceptibility test using a Mueller-Hinton agar plate, the presence of extended-spectrum beta-lactamase (ESBL) negativity, sensitivity to ciprofloxacin, intermediate sensitivity to tigecycline and resistance to ampicillin/sulbactam, piperacillin/tazobactam, cephalosporins (cefoxitin, ceftazidime, ceftriaxone and cefepime), carbapenems (meropenem, imipenem, ertapenem and doripenem) and aminoglycosides (amikacin and gentamicin) were reported.

The results of these tests were as follows:

**Table:** 

Antibiotics	MIC	Sensitivity
Ampicillin/Sulbactam	>=32	Resistant
Piperacillin/Tazobactam	>=128	Resistant
Cefoxitin	>=64	Resistant
Ceftazidime	32	Resistant
Ceftriaxone	>=64	Resistant
Cefepime	>=64	Resistant
Meropenem	>=16	Resistant
Imipenem	4	Resistant
Ertapenem	>=8	Resistant
Doripenem	>=8	Resistant
Amikacin	>=64	Resistant
Gentamicin	>=16	Resistant
Tigecycline	4	Intermediate sensitivity
Ciprofloxacin	0.5	Sensitive

We then decided to start treatment with ciprofloxacin for 10 days with a favorable response, with resolution of the pneumonic process after 10 days of antibiotics.

## Discussion

This case presents an opportunity to report emerging microorganisms in our country and represents an opportunity to detect new agents in the field of infections associated with healthcare. Compared to the reviewed bibliographies, our case resembles the reports of other countries and agrees with the characteristics referred by them; therefore, we would improve the information available about this etiological agent and the treatments used to eradicate these infections.

Our case closely resembles the clinical presentation reported in the literature, with the patient being a female, with multiple comorbidities that, after requiring mechanical ventilation, develop ventilator-associated pneumonia. It is also important to point out that the incidence in hospitals in our region is 0, and this case represents the first appearance of a new microorganism in our region, so it is important to report its clinical and microbiological characteristics to start considering it as a causative agent of intrahospital pneumonia. Within its microbiological characteristics, our case perfectly agrees with the appearance in Blood Agar of an extremely mucous bacterial culture after the reported incubation time and requiring a nonempirical treatment in this case ciprofloxacin as in those reported in the literature that this antibiotic was used after having an antibiogram that showed susceptibility to it.

## Conclusion

We must start to consider new etiological agents associated with nosocomial infections, which are identified thanks to new technologies, such as molecular techniques, allowing us to update the treatment guidelines and learn which are the most frequent bacteria in our country. This is the first case reported in the state of Veracruz and the second in Mexico. We should consider that this pathogen affects not only adults with multiple comorbidities but also children with renal, hepatic, or oncological pathologies, as well as immunocompromised patients, who should be considered high-risk populations for W. virosa infection. The identification of new pathogens by using molecular techniques is a great opportunity to expand epidemiological surveillance and establish new guidelines for empirical antibiotic therapies.

More clinical information, diagnosis and treatment of this rare pathogen is needed. A review of the literature from 1990 to 2022 found only 13 cases with Weeksella virosa infections.(Table 1). We found that these cases presented with the following clinical syndromes: pneumonia (2/13), spontaneous bacterial peritonitis (2/13), sepsis (3/13), urinary tract infection (2/9), pneumonia (2/13), infection of a surgical head wound with ventriculitis (1/13), chorioamnionitis (1/13) and surgical wound infection (1/13). Nine of the 13 cases, including ours, have been found in the last 10 years, which interestingly shows an increase in the incidence of infection by this bacterium in humans. Currently, no risk factor directly involved has been established, but all patients who presented with this infection presented at least one comorbidity, such as diabetes mellitus (3/13), end-stage renal disease (5/13), hepatitis C virus infection (1/13), ischemic heart disease (1/13), lymphoma (1/13) or anaplastic meningioma (1/13) [16].

**Table 1:** Case reports confirmed Weeksella virosa infection

Author	Year of Publication	Age	Sex	Comorbidities	Origin	Clinical Syndrome	Antimicrobial Susceptibility	Treatment	Outcome
Faber et al. [9]	1991	33	F	Terminal Kidney Failure	Peritoneal Liquid	Spontaneous Bacterial Peritonitis	S: Ampicillin, Ceftizoxime, Mezlocillin and Imipenem/Cilastatin R: Aminoglycosides, Trimethoprim and Sulfamethoxazole, Ciprofloxacin, Cefazolin, Cefotetan and Ceftazidime	Imipenem/Cilastatin	Survived
Boixeda et al. [10]	1998	55	M	Hepatitis C Virus Infection/Cirrhosis of the Liver	Peritoneal Liquid	Spontaneous Bacterial Peritonitis	Sensitivity to Cefoxitin	Cefoxitin	Survived
Meharwal et al. [11]	2002	Not Reported	Not Reported	Not Reported	Urine	Urinary tract infection	None Reported	None Reported	Unreported
Manoragan et al. [8]	2004	53	F	Lymphoma/Diabetes Mellitus/Terminal Renal Failure in Hemodialysis	Blood/Sputum	Pneumonia	S: Cefepime, Ceftazidime, Ceftriaxone, Piperacillin and Imipenem/Cilastatin. R: Ciprofloxacin, Levofloxacin, Tobramycin, Trimethoprim with Sulfamethoxazole and Gentamicin.	Cefepime/Vancomycin	Passed away
Slenker et al. [15]	2012	44	F	Obesity/Menorrhagia	Wound	Labial Wound Infection	S: Aztreonam, Meropenem and Piperacillin R: Amikacin, Gentamicin, Tobramycin, Ciprofloxacin, and Trimethoprim with Sulfamethoxazole	Incision and Drainage	Survived
		31	F	Ischemic Heart Disease/Terminal Renal Failure/AMI/Smoking/Asthma/Hepatitis C Virus Infection/Obesity	Blood	Sepsis/Bacteremia	S: Aztreonam, Ceftazidime, Gentamicin, Meropenem, Piperacillin and Imipenem/Cilastatin R: Amikacin, Ciprofloxacin, and Trimethoprim with Sulfamethoxazole	Aztreonam/Tobramycin	Passed away
		25	F	Spontaneous Vaginal Delivery	Placenta	Amnionitis	S: Aztreonam, Ampicillin, Meropenem and Piperacillin R: Amikacin, Tobramycin, Ciprofloxacin and Trimethoprim with Sulfamethoxazole	Ampicillin/ Gentamycin	Survived
		26	F	Endometriosis/Abdominopelvic Adhesiolysis/Diabetes Mellitus	Urine	Urinary Tract infection	S: Aztreonam, Meropenem and Piperacillin R: Amikacin, Gentamicin, Tobramycin, Ciprofloxacin	Trimethoprim with Sulfamethoxazole	Survived
Toescu et al. [12]	2017	50	F	Anaplastic Meningioma/Use of Glucocorticoids/ Radiation therapy	Head Injury/ Cerebral Ventricle	Surgical Wound Infection (Craniotomy)/Ventriculitis	S: Amoxicillin with Clavulanate, Cephalosporins, Meropenem and Piperacillin-Tazobactam R: Gentamicin and Ciprofloxacin	Ceftriaxone/Amoxicillin	Survived
Cowgirl et al. [16]	2019	4	M	Embryonic Rhabdomyosarcoma	Blood	Bacteremia	S: Amikacin, Gentamicin, Ampicillin, Cefuroxime, Ciprofloxacin, Piperacillin/Tazobactam and Tetracyclines. R: Aminoglycosides	Imipenem/Meropenem	Survived
Unalan et al. [14]	2019	4	F	Addison’s Disease and Terminal Renal Failure with Peritoneal Dialysis	Peritoneal liquid	Peritonitis Associated with Peritoneal Dialysis Catheter	S: Imipenem, Meropenem, Piperacillin I: Amikacin and Ciprofloxacin R: Cephalosporins.	Meropenem and Removal of Catheter	Survived
Campbell et al. [18]	2020	Newborn	Not Reported	Extreme Premature (26 SDG) with Low Weight Extreme at Birth and Spontaneous Vaginal Delivery.	Blood	Early Neonatal Sepsis	S: Ampicillin and Meropenem I: Ceftazidime R: Cefepime and Ceftriaxone	Meropenem	Survived
Current Report	2022	64	F	Post-Cardiac Arrest Syndrome, Diabetes Mellitus, Renal Chronic Disease KDIGO III, Soft Tissue Infection in Left Pelvic Limb, Mechanical Ventilation.	Blood/Sputum	Pneumonia by Mechanical Ventilation	S: Ciprofloxacin I: Tigecycline R: Ampicillin/Sulbactam, Piperacillin-Tazobactam, Cephalosporins, Carbapenems and Aminoglycosides.	Ciprofloxacin	Survived

F: Female M: Male

S: Susceptible I: Intermediate Susceptibility R: Resistant

Modified from (Vaquera-Aparicio et al. [16])

## Data Availability

The data sets generated and/or analyzed during this study are not publicly available because they are owned by a federal public hospital but are available from the corresponding author upon reasonable request.
